# Provision of inadequate information on postnatal care and services during antenatal visits in Busega, Northwest Tanzania: a simulated client study

**DOI:** 10.1186/s12913-022-08071-6

**Published:** 2022-05-25

**Authors:** Eveline T. Konje, Itikija E. Msuya, Dismas Matovelo, Namanya Basinda, Deborah Dewey

**Affiliations:** 1 Department of Biostatistics and Epidemiology, School of Public Health, Catholic University of Health and Allied Sciences, Mwanza, Tanzania; 2 Department of Obstetrics and Gynecology, School of Medicine, Catholic University of Health and Allied Sciences, Mwanza, Tanzania; 3 Department of Community Medicine, School of Public Health, Catholic University of Health and Allied Sciences, Mwanza, Tanzania; 4 Departments of Pediatrics and Community Health Sciences, Cumming School of Medicine University of Calgary, Calgary, AB Canada; 5 Owerko Centre at the Alberta Children’s Hospital Research Institute, University of Calgary, Calgary, AB Canada; 6 Hotchkiss Brain Institute, University of Calgary, Calgary, AB Canada

**Keywords:** Antenatal care, Postnatal care, Practices of health care workers, Postnatal education, Simulated client

## Abstract

**Background:**

Most (94%) of global maternal deaths occur in low- and middle-income countries due to preventable causes. Maternal health care remains a key pillar in improving survival. Antenatal care (ANC) guidelines recommend that pregnant women should be provided with information about postnatal care in the third trimester. However, the utilization of postnatal care services is limited in developing countries including Tanzania. The aim of this study was to investigate the practice of health care workers in providing information on postnatal care to pregnant women during antenatal care visits.

**Methods:**

A cross sectional study was conducted among health care workers from 27 health facilities that offer reproductive and child health services in Busega district Northwest Tanzania. A simulated client approach was utilized to observe quality of practice among health care workers with minimal reporting bias (i.e., the approach allows observing participants at their routine practices without pretending). Selected pregnant women who were trained to be simulated clients from the community within facility catchment area attended antenatal care sessions and observed 81 of 103 health care workers. Data analyses were carried out using STATA 13.

**Results:**

Only 38.73% (95% CI; 28.18–49.49%) of health care workers were observed discussing subtopics related to postnatal care during the ANC visit. Few health care workers (19.35%), covered all eight subtopics recommended in the ANC guidelines. Postnatal danger signs (33.33%) and exclusive breast feeding (33.33%) were mostly discussed subtopics by health care workers. Being a doctor/nurse/clinical officer is associated by provision of postnatal education compared to medical attendant, aOR = 3.65 (95% CI; 1.21–12.14).

**Conclusion:**

The provision of postnatal education during ANC visits by health care workers in this district was limited. This situation could contribute to the low utilization of postnatal care services. Health care workers need to be reminded on the importance of delivering postnatal education to pregnant women attending ANC clinic visits. On job training can be used to empower health care workers of different cadres to deliver postnatal health education during ANC visits. These efforts could increase women’s utilization of postnatal care and improve outcomes for mothers and newborns.

**Supplementary Information:**

The online version contains supplementary material available at 10.1186/s12913-022-08071-6.

## Background

Despite international and national efforts to reduce maternal and newborn mortality [1, 2], women and their newborns from poor countries still experience life threatening conditions [3, 4]. In 2017, it was estimated that 295000 maternal deaths occurred during pregnancy, delivery, and postnatal with 94% reported from low- and middle-income countries (LMICs) [5]. In 2019, around 6700 newborns died every day globally [6]. In Tanzania, maternal mortality rate remains unacceptably high at 524 per 100,000 live births as well as neonatal mortality rate at 25 per 1000 live births [5]. In LMICs including Tanzania, most maternal deaths occur within the first 24 h after delivery and up to 75% of neonatal deaths occur within the first week of life [5]. The majority of maternal and neonatal deaths are due to preventable conditions such as hemorrhage, sepsis, hypertensive disorders, or neonatal sepsis, birth asphyxia and prematurity, respectively [5, 7–9]. Antenatal care is one of the pillars in the Safe Motherhood Initiative for promoting and improving maternal and child health through health education, early detection and management to mention a few. The provision of quality ANC to pregnant women has been associated with improved facility health seeking behavior during childbirth and postnatal [10]. The postnatal period carries a high risk of mortality and morbidity for women and their newborns [11, 12]. However, the provision and utilization of postnatal care services is limited in LMICs countries [13–15]. One factor that could influence women’s ultization of these services is the lack of or limited information provided about these services during antenatal care (ANC). The poor uptake of postnatal care in developing countries may impede the global effort to reduce preventable maternal deaths to 70 per 100000 live births and neonatal mortality to less than 12 per 1000 live births as per sustainable development goal 3; targets 3.1 and 3.2 by 2030 [16]. Maternal and child health services are the key pillars in preventing maternal and newborn deaths and improving survival.

The ANC guidelines recommend that pregnant women be provided with information about postnatal care and available services in the third trimester and at discharge after delivery [17]. However, there has been a limited change in postnatal care uptake among women and their newborns in Tanzania over the past decade. In 2010, 35% of women and their newborns utilized postnatal care (PNC) services, whereas in 2016 only 37% used PNC services. This is in contrast to the almost universal utilization (98%) of ANC services with one half of women (51%) attending at least four ANC visits [18].

According to the ANC guidelines, health care providers should educate pregnant women on: 1) postnatal danger signs for both themselves and their newborns, 2) the importance of postnatal visits with health care providers, 3) proper nutrition during postnatal period, 4) exclusive breast feeding, 5) postnatal family planning, 6) prevention of disease particularly malaria, 7) personal hygiene, and 8) other health practices such as cord care, immunization, eating habits, and birth registration. Research has reported that such educational practices positively impact newborn care practices and health care seeking behavior [13–15, 19, 20]. The limited number of studies conducted in Tanzania that have examined the educational practices of health care workers during ANC visits have reported that they tend to focus only on danger signs during pregnancy, birth preparedness information, and breastfeeding practices [20–22]. Other components, particularly those related to postnatal care are not discussed or only mentioned occasionally. Therefore, in the present study, we aimed to investigate the practices of health care workers in providing information on postnatal care to pregnant women during ANC visits using simulated client method in Busega district Northwest Tanzania.

## Methods

### Study setting

Simiyu region is located in Lakezone Northwest Tanzania. Busega is one of six districts located in Simiyu Region Northwest Tanzania. It was purposively selected due to the low uptake of maternal and child health services [18]. Based on the Tanzania Demographic and Health Survey, Simiyu was reported to be the lowest region in the country for utilization of maternal and child health services; 60% of pregnant women deliver at home and only 10% of women attend postnatal care services [18]. Busega district has 29 health facilities. During the study period, 27 health facilities offered reproductive and child health services (RCH); 22 were public health facilities (District Health Information System, DHIS2). Of 27 facilities that participated in the study, two were hospitals, four health centers, and 21 dispensaries.

### Study design

A cross-sectional study that used a simulated client method was conducted between August and September 2020 in all health facilities that provided RCH services in Busega district Northwest Tanzania.

### Sample size and procedure

Busega district has 251 health providers with different medical qualifications and skills (i.e. medical doctors, assistant medical officers, registered/enrolled nurses, clinical officers, assistant clinical officers, medical attendants) with approximately 40% (~ 103) working in RCH units. Using the Yamane Taro formula for a finite population [23] and a tolerable error of 0.05, we estimated that a sample of 81 health care workers was required to address our research question. As a result, more than 75% of all health workers in RCH units in this district were involved in this study. Any health care worker who provided service in RCH clinic during the study period was included. To avoid investigating the same health worker more than once over the study period, the monthly roster of each facility was obtained from the facility director and used to plan the simulated client visits. Three health care workers from each of 27 participating facilities were involved. To avoid suspicion among health care workers, a simulated client attended antenatal care clinic once for only one health facility. Hence, three simulated clients visited one health facility each on a different day, over three consecutive days. Health care workers were blinded to the study to minimize potential biases that could result in changes in their provision of postnatal care education to pregnant women due to prior knowledge of the study.

### Simulated client method

A simulated client method is one means of studying client-provider interactions. It has been used to evaluate or assess the quality of services or care provided by health worker’s in family planning programs and antibiotic dispensing practices [21]. It consists of sending clients, who do not reveal their identity to a service provider to receive a particular service and interviewing them after the encounter. This approach minimizes reporting and observation biases that could be introduced by face-to-face interviews or observational data collection methods [21, 22]. This approach was approved by ethical committee. In this study, the simulated clients were pregnant women in their 3^rd^ trimester who had attended ANC clinics for services. Pregnant women who lived near the health facility were purposively identified through the ANC register, and three were randomly selected to participate as simulated clients in the study. Women with no known medical conditions that could complicate their pregnancies were invited to participate in this study as simulated clients. A detailed medical history was obtained from the women by trained research assistants (i.e., medical doctors who were not health workers in the study area) to ensure that they had no known medical problems before engaging them as simulated clients. To ensure uniform presentation and consistency of data collection, the simulated clients were trained for one day on how to behave as a client. Information on the topics that should be covered by health workers regarding postnatal care (i.e., postnatal danger signs, importance of completing postnatal visits, proper nutrition during postnatal period, infant feeding practices with emphasis on exclusive breast feeding, postnatal family planning, self-care, hygiene, and newborn care) were reviewed with the simulated clients during this training [12, 24].

### Data collection procedure

Back and forth translation of the structured interview data collection tool from English to Kiswahili to English was done by two research assistants to maintain consistency of the questions prior to data collection activities (see supplementary files). A structured interview was piloted with ten pregnant women who were not simulated clients prior to data collection to ensure that women understood the questions and had no difficulty in answering them. All interviews were conducted in Kiswahili which is a national language and recorded in the structured questionnaire.

On the data collection day, immediately after attending ANC appointment, a structured face-to-face interview (see supplementary file) was conducted with the simulated client by a trained research assistant. This interview took place in the village leader's office after the client left the health facility premise. The structured interview focused on the postnatal education topics noted in the previous section were discussed by the health care worker during the ANC visit. Probing questions were also asked for “yes” response to all eight subtopics if they were mentioned during antenatal session as a quality check (validating questions) to minimize guess response by simulated clients. However, we acknowledged the difference in recall ability of simulated clients. To accommodate this, the failure to respond correctly to follow up question did not alter the previous “yes” response.

A questionnaire completed by the director/in charge of each health facility captured information on the characteristics of the health workers including age, sex, level of qualification and work experience in the RCH unit. For this study, health care providers were classified as skilled (i.e., medical officer, assistant medical officer, nurses) or unskilled (i.e., medical attendants) based on the level of medical training; a medical officer is trained for five years, a clinical officer is trained for three years, an assistant nursing officer receives two years of training, an enrolled nurse also receives three years of training, while medical attendants receive one year of training.

### Data management and statistical analysis

As per Tanzania National ANC guidelines, health workers are to provide education on postnatal care to pregnant women in their 3^rd^ trimester [24]. Ideally, all eight components in postnatal care listed in the previous section should be discussed with pregnant women. If any of the eight topics was discussed to pregnant women during antenatal care session, health worker was considered providing postnatal care education. Zero score was applied when none of the eight components were discussed by health care workers whereas a score of one was given when any component was discussed. Regardless of the topic, minimum of four components were discussed by health care workers in this study. This was used to show the extent of practice in the provision of postnatal care education to pregnant women during antenatal care session. The minimum number of components (i.e., four topics) was used to classify health care workers either practice in the provision of postnatal education or not. Hence in this study, the reported practice variable was considered based on the scoring system at the cutoff of 4. Those who scored four or above were classified as health care workers providing health education whereas those with less scores were classified as not providing postnatal education during ANC sessions. This was a key binary variable for the study.

Data were entered in Ms-Excel and analyzed using STATA version 13. Socio-demographic characteristics of the health workers and health facilities profiles were summarized using descriptive statistics. The median and interquartile range were considered for skewed continuous variable i.e., age of participants whereas percentage was used for categorical variables. The associations between the provision of postnatal education and other factors including level of health facility, age, sex, work experience (i.e., length of time working in RCH unit), and level of qualification of health care providers (i.e., skilled vs unskilled) were investigated using chi-square tests. With limited sample size in this study, an exact binary logistic regression was performed to investigate the associations between the provision of postnatal education by health workers and factors such as level of health facility, skill level, sex, and age. We used exact logistic regression as an alternative estimator to the maximum likelihood logistic to overcome overestimation in odds ratios for studies with small to moderate samples size [25, 26]. At bivariate analysis, each variable was associated with outcome of interest (practice) and variables that were found to be significant at the bivariate analysis were included in the multivariate analysis using p value of less than 0.05. Backward elimination procedure was used to remove variable from the model. All variables that were significant at bivariate were included in the initial model and removed when p value was greater than 0.05. The procedure was repeated to retain only significant (p value less than 0.05) variables in the model. Odds ratios with 95% confidence intervals were estimated for both bivariate and multivariate associations.

## Results

### Socio-demographic characteristics of health providers

A total of 81 health providers were observed by simulated clients for their practice in providing postnatal care education to pregnant women at ANC sessions. Three-quarters of the health care providers (76.54%) were female and the median age was 33 years (IQR, 30–36 years). The majority of health care providers were assistant nursing officers, clinical officers or enrolled nurses who were classified as skilled (60.49%). Almost all of the health care providers (90.12%) had worked at RCH unit for at least one year. See Table 1

**Table 1:** Socio-demographic characteristics of the Health Care Workers (*N* = 81)

Variable	Frequency(n)	Percentage (%)
**Health providers’ characteristics (*N* = 81)**		
Sex		
Male	19	23.46
Female	62	76.54
Qualification/medical cadre		
^a^Skilled	49	60.49
^b^Unskilled	32	39.51
Duration working in RCH clinic		
< 1 year	8	9.88
1–3 years	73	90.12

^a^ skilled (medical officer, assistant medical officer, nurses)

^b^ unskilled (medical attendants)

### Provision of postnatal care education based on simulated clients

Only 38.73% (95% CI; 28.18, 49.49%) of the health workers observed to discuss at least any of the eight topics of postnatal care during ANC visits. Four was the least number of topics discussed with pregnant women. However, very few health care workers (7.41%) covered all eight topics. The most commonly discussed postnatal care topics were postnatal danger signs (33.33%) and exclusive breast feeding (33.33%). Mother and baby hygiene (23.46%), and self-care and other health practices such as cord care, immunization, eating habits, and birth registration were not commonly discussed (20.99%). See Table 2.

**Table 2:** Reported practice of providing postnatal education during ANC by simulated clients based on topic taught (*N* = 81)

Variable	HC providers	Percent (%)
**Number of topics taught**		
•No topic discussed	50	61.73
•One topic discussed	0	-
•Two topics discussed	0	-
•Three topics discussed	0	-
•Four topics discussed	4	4.94
•Five topics discussed	10	12.35
•Six topics discussed	7	8.64
•Seven topics discussed	4	4.94
•Eight topics discussed	6	7.41
**List of topics that were taught by HCP**^a^ **who could have discussed more than one topic**		
•Postnatal danger signs	27	33.33
•Infant feeding (exclusive breast feeding)	27	33.33
•Importance of attending postnatal visits as per schedule and completion of postnatal visits	26	32.10
•Proper nutrition for you and your baby during postnatal period	24	29.63
•Postpartum family planning and its importance	22	27.16
•Prevention of disease particularly malaria during postnatal period	22	27.16
•Personal hygiene for you and your baby during postnatal period	19	23.46
•Self-care and other health practices during postnatal period	17	20.99
**General practice on provision of postnatal education**		
•Those who discussed 0–3 topics (not practiced)	50	61.73
•Those who discussed 4–8 topics (practiced)	31	38.27

^a^ *HCP* health care provider

### Factors associated with provision of postnatal education during antenatal ANC visits

We assessed the facility and socio-demographic characteristics associated with health care workers’ practice of discussing postnatal care with pregnant women during antenatal sessions. Health workers’ age and sex were not associated with provision of postnatal care education to pregnant women during ANC visits. Similarly, number of years working at the RCH clinic was not associated with provision of postnatal education to pregnant women. In this study, being skilled or unskilled health personnel was associated with provision of postnatal education during antenatal session with OR 4.28; 95% CI, 1.45–14.06. The odds of discussing postnatal care issues with pregnant women was four times higher among skilled health workers (medical officer, assistant medical officer, nurses compared to unskilled personnel (medical attendants). Level of facility was also significantly association with the practice of healthcare providers (OR 3.32, 95% CI, 1.01–11.78). That is, the odds of discussing postnatal care topics was three times higher among health care workers at hospitals/health centers compared to those at dispensaries. In the multivariable analysis, only heath care workers’ level of skill was significantly associated with provision of postnatal education during antenatal visits (AOR 3.65, 95% CI 1.21–12.14). Skilled health care workers had an almost four times higher odds of providing postnatal care education to pregnant women at ANC clinics than unskilled workers (see Table 3).

**Table 3:** Factors associated with provision of postnatal care education during ANC session

	**Bivariate analysis**		**Multivariate analysis**	
**Variable**	**OR(95%CI)**	***P*-Value**	**AOR(95%CI)**	***P*-Value**
**Age group**				
> 33 years	1			
< = 33 years	1.06 (0.38–2.95)	1.000		
**Sex**				
Female	1			
Male	1.23 (0.37–3.94)	0.893		
**Health workers qualification**				
Unskilled	1		1	
Skilled	4.28 (1.45–14.06)	0.006	3.65 (1.21–12.14)	0.019
**Duration working at RCH clinic**				
< one year	1			
> = one year	1.03 (0.18–7.19)	1.000		
**Facility ownership**				
Public	1			
Private	1.09 (0.28–3.93)	0.879		
**Level of facility**				
Dispensary	1		1	
Hosp/Health center	3.32 (1.01–11.78)	0.028	2.54 (0.72–9.44)	0.169

^*^ *RCH* reproductive & child health

## Discussion

The study investigated the practice of RCH health care workers in providing postnatal care education to women at ANC visits in the 3^rd^ trimester in Busega, Simiyu Region Northwest Tanzania. In this setting, we found that only 38% health workers provided postnatal care education (i.e., discussing any of the eight components of postnatal care identified in the ANC guidelines) to pregnant women during prenatal appointments. Almost two third (62%) of health workers provided no postnatal education. Only few health providers discussed the eight recommended components that are to be delivered as part of the postnatal education package to pregnant women. Information on postnatal danger signs (33%), exclusive breastfeeding (33%), maternal and infant nutrition (30%), and family planning (27%) were the most common components discussed by health workers. Unskilled health care workers were less likely to educate women about postnatal care compared to skilled health care workers.

Previous research that has examined the provision of postnatal care information to women by health workers in LMICs has revealed significant variability in the topics that are discussed with some rarely mentioned and others commonly discussed during ANC visits. A study by Gross et al. in Morogoro, Tanzania that used a participant observation technique found low provision of health education in the areas of family planning (8%), breastfeeding (11%), and hygiene (28%) compared to birth plan counselling (50%) [27]. In other countries such as Burkina Faso and Uganda a limited number of health workers discussed with pregnant women on maternal and infant nutrition (2% vs. 31%,) and postnatal danger signs (2% vs 7%), respectively [28]. In Gambia, only about one-third of pregnant women were provided with health education on diet and nutrition (35%) and baby care (30%), and one quarter on family planning (24%) [29]. In contrast, studies by Duysburgh et al. and Conrad et al. in Tanzania reported that greater than 50% of health providers discussed with pregnant women on postnatal danger signs and nutrition [30, 31]. Discrepancies in the findings of these studies could be due to methodological differences. The present study used a simulated client approach in which health care providers were blinded to the study and its objectives, whereas other studies have used direct observation or exit interviews with health workers. Direct observation and interviews with health workers may introduce bias since the providers are aware of the study. Hence, they may change in their practice or report educational activities that differ from their typical practice due to awareness of study, which could result in an over estimation of the postnatal education provided to pregnant women during ANC clinic appointments.

Limited practice in the provision of postnatal care education by health care workers to pregnant women during ANC sessions has been reported to be due to inadequate staffing levels, inadequate knowledge on the part of health care providers, and poor infrastructure at health facilities, including the lack of necessary resources such as teaching materials and ANC guidelines [13, 27, 32]. In the present study, the poor provision of postnatal education during ANC could partially explain the low utilization of postnatal care observed in this region, and the persisting high mortality rates among women and their newborns [18].

ANC services are used by almost all pregnant women in Tanzania [18] and health education and counselling during the antenatal period can reduce pregnancy and postnatal complications [33, 34]. ANC clinics provide health workers with the opportunity to discuss risks to maternal and newborn health and healthy behaviors during pregnancy and postpartum with pregnant women. Studies that have examined risk factors associated with maternal mortality suggest that low levels of awareness of post-delivery problems contribute to maternal mortality and neonatal mortality [35–37]. Therefore, during ANC visits, health care providers need to assist their clients in understanding how symptoms and behaviors in the postnatal period are related to clinical outcomes for themselves and their newborns. This could assist women in recognizing and acting on danger signs earlier, accelerating the timeline of referrals, and thus avoiding the adverse consequences associated with a potentially serious conditions. Self-identification of postnatal danger signs and other health issues related to the postnatal period by women themselves and early reporting to health facilities could reduce maternal and neonatal deaths. A well-organized postnatal education program during ANC visits could increase knowledge and change the attitudes of pregnant women regarding the importance of postnatal care for themselves and their newborns, which may eventually increase utilization of these services and reduce maternal and newborn mortality.

### Strengths and limitations of the study

Our study has some strengths including the inclusion of over 75% of the health care providers who worked in RCH in Busega district and the inclusion of all health facilities that offered RCH services in Busega district. Therefore, a majority of the health care workers involved in RCH in this district were represented. Other strengths of the study are the use of simulated clients who observed and reported on the information that health workers provided to pregnant women on postnatal care while blinding them of the study. This methodology allowed us to obtain a valid estimate of the health care workers’ practice that was not biased by prior knowledge of the study, which may have changed the practice of these health care providers. This study also has some limitations. Since the study was conducted in only one district in Tanzania, the results may not be generalized to other settings. Furthermore, use of simulated clients from the same community where health worker resided had the potential to introduce bias as we cannot rule out that there was no communication between the simulated clients and health workers that may have resulted in a change in practice. Since the simulated clients were regular users of the facility, the findings could be influenced by how they perceive the services at the facility rather than the actual performance of the health care workers. However, this bias is expected to be minimal after conducting training among pregnant women on how to behave as simulated clients as well as reporting of what was observed rather than what they know and perceive prior to data collection. Lastly, the inclusion of on job training, supervision, health workers’ workload, and availability of teaching aids would shed lights on limited health workers’ practices in provision of PNC education.

## Conclusion

More than half of the health care workers were not providing postnatal education during ANC visits to pregnant women in Busega district, Simiyu region. This practice could be associated with low utilization level of postnatal care services in this area as women may not be well-informed about available services post-delivery. There is a need for supportive supervision, on job training or refreshers course to empower health workers with relevant knowledge and skills on postnatal care education. Furthermore, due to shortage of skilled health care workers, medical attendants and community health care workers can be educated and empowered to provide postnatal care education to pregnant women with minimal supervision. Strengthening postnatal education during ANC visits for pregnant women attending ANC clinics by providing important and relevant information and employing teaching aids could ensure increased utilization of postnatal care and result in improved outcomes for mothers and newborns.

## Supplementary Information

**Additional file 1: Supplementary file. **Questionnaire (English version).

**Additional file 2: Supplementary file. **Questionnaire (Kiswahili version).

## Data Availability

The dataset and research materials from which conclusions are drawn are available upon request from the corresponding author.

## Abbreviations

ANC: Antenatal Care
AOR: Adjusted Odds Ratio
CI: Confidence Interval
CUHAS: Catholic University of Health and Allied Sciences
DHIS: District Health Information System
HCP: Health care provider
LMICs: Low and Middle Income Countries
OR: Odds Ratio
PNC: Postnatal Care
RCH: Reproductive and Child Health
