# Supplementary material for: Provision of inadequate information on postnatal care and services during antenatal visits in Busega, Northwest Tanzania: a simulated client study
Source: BMC Health Serv Res. 2022 May 25;22:700. doi: 10.1186/s12913-022-08071-6 (PMC9131525; doi:10.1186/s12913-022-08071-6)
Supplement: Supplementary file 1 — Additional file 1: Supplementary file. Questionnaire (English version). [file 12913_2022_8071_MOESM1_ESM.pdf]

Supplementary file: Questionnaire (English version)

Section 1: Facility's information

SN	PARTICULAR	RESPONSE
1.1	Facility Name (mention please)	
1.2	Owner of Facility; 1 = Government, 2 =Private	[ ]
1.3	Type of Facility 1 = Dispensary, 2 = Health Center, 3 = Hospital	[ ]

Section 2: Demographic characteristics of health care provider [obtain from in charge]

2.1	Health worker's age (years)	[ ]
2.2	Health worker's sex: 1=Male, 2= Female	[ ]
2.3	Level of skill: 1=skilled 2=unskilled<br>Skilled(doctor, clinical officer, nurse)Unskilled (medical attendants)	[ ]
2.4	How long have [participant] worked in RCH clinic?<br>1 = < 1 year, 2 = 1 -3 years	[ ]

Section 3: Practices on provisions of postnatal

S/N	Question	probe
3.1	Education about postnatal danger signs	Did health provider taught you? If Yes,<br>What have you taught, can you tell us some of postnatal danger sign?
3.2	The important of attending postnatal visits as per schedule and to complete postnatal visits	Did health provider taught you or mention to you importance of postnatal schedules? If Yes,<br>How many visit are you required to attend?
3.3	Education on postpartum family planning and its important;	Did health provider taught you about postpartum family planning? If Yes, can you mention some methods mentioned by health provider?
3.4	Education on Infant feeding focus on exclusive breast feeding	Did health provider taught you about exclusive breast feeding? If Yes, can you tell me the meaning of exclusive breast feeding and its importance
3.5	Education on person hygiene for you and your baby during postnatal period	Did health provider taught you about hygiene for you and your baby? If Yes, can you explain the meaning of person hygiene, can you mention one advantages
3.6	Education on prevention of diseases particularly Malaria during postnatal period	Did health provider taught you about prevention of malaria? If Yes, can you mention on how do you prevent malaria?
3.7	Education on self-care and other healthy practices during postnatal period	Did health provider taught you about self-care and other healthy practices during postnatal period? If Yes, can you mention how you care for your baby and yourself?
3.8	Education on proper nutrition for you and your baby during postnatal	Did health provider taught you about proper nutrition for you and your baby during postnatal? If Yes, can you briefly tell me proper nutrition for your baby and yourself?
