# Supplementary material for: Provision of inadequate information on postnatal care and services during antenatal visits in Busega, Northwest Tanzania: a simulated client study
Source: BMC Health Serv Res. 2022 May 25;22:700. doi: 10.1186/s12913-022-08071-6 (PMC9131525; doi:10.1186/s12913-022-08071-6)
Supplement: Supplementary file 2 — Additional file 2: Supplementary file. Questionnaire (Kiswahili version). [file 12913_2022_8071_MOESM2_ESM.pdf]

Supplementary file: Questionnaire (Kiswahili version)

1. **Sehemu ya kwanza: Taarifa za kituo cha huduma**

1.1	Jina la kituo (Taja tafadhali)	[ ]
1.2	Mmiliki: 1 = Serikali 2 = Binafsi	[ ]
1.3	Aina ya kituo: 1 = Zahanati, 2 = Kituo cha Afya 3 = Hospitali	

2. **Sehemu ya pili: Taarifa za mtoa huduma za Afya**

2.1	Umri wa mtoa huduma (miaka)	[ ]
2.2	Jinsia ya mtoa huduma: 1=Me, 2= Ke	[ ]
2.3	Aina ya kada/kiwango cha ujuzi<br>1. Mjuzi (Daktari, muuguzi, muuguzi msaidizi, afisa tabibu, tabibu msaidizi)<br>2. Sio mjuzi (muhudumu msaidizi wa afya)	[ ]
2.4	Amefanya kazi kwa muda gani katika kiliniki ya afya ya mama na mtoto 1 = < mwaka, 2 = 1 – 3 miaka	[ ]

3. Sehemu ya tatu: Utoaji wa elimu ya kipindi cha uzazi

3.1	Elimu kuhusu dalili za hatari wakati wa kipindi cha uzazi	Je umefundishwa hii maada? Kama ndiyo, unaweza kutuambia dalili za hatari wakati wa kipindi cha uzazi?
3.2	Umuhimu wa kuhudhuria kliniki baada ya kujifungua kulingana na muongozo na kumaliza mahudhurio yote manne	Je umefundishwa umuhimu wa kuhudhuria kliniki kipindi cha uzazi? Kama ndiyo, je unatakiwa kuhudhuria mara ngapi?
3.3	Elimu juu ya uzazi wa mpango wakati wa kipindi cha uzazi	Je umefundishwa kuhusu kuhusu mpango wa uzazi katika kipindi cha uzazi?Kama ndiyo, unaweza ukatuambia baadhi ya njia salama zilizotajwa na mtoa huduma?
3.4	Elimu juu ya kumlisha mtoto maziwa ya mama peke na umuhimu wake	Je ulifundishwa kuhusu kumlisha mtoto maziwa ya mama peke yake na umuhimu wake? Kama ndiyo, nini maana yakumlisha

		mtoto maziwa ya mama tu, na umuhimu wake?
3.5	Elimu juu ya usafi wako na wa mtoto katika kipindi cha uzazi	Je ulifundishwa kuhusu elimu ya usafi wako nawa mtoto katika kipindi cha uzazi? Kama ndiyo, unaweza elezea maana ya usafi wa mwili au binafsi?, je unaweza taja faida moja ya usafi katika kipindi cha uzazi?
3.6	Elimu jinsi ya kujikinga na magonjwa hasa malaria katika kipindi cha uzazi	Je umepata elimu ya kujikinga na magonjwa hasa malaria? Je unaweza taja ni kwa jinsi gani unaweza kujikinga na malaria?
3.7	Elimu jinsi ya kujiweka katika mazingira salama naya kiafya katika kipindi cha uzazi	Je umepata elimu ya kujiweka katika mazingira salama naya kiafya katika kipindi cha uzazi? Kama ndiyo, unaweza kutaja jinsi ya kumjali mtoto wako au mwenyewe?
3.8	Elimu juu ya lishe bora kwako na kwa mtoto katika kipindi cha uzazi	Je umepata elimu juu ya lishe bora kwako na kwa mtoto kipindi cha uzazi? Kama ndiyo, kwa kifupi nieleze lishe bora ni nini?
